# *catena*-Poly[[bis­(μ-2-formyl-6-meth­oxy­phenolato)copper(II)sodium]-μ-nitrato]

**DOI:** 10.1107/S1600536811040025

**Published:** 2011-10-12

**Authors:** Po Gao, Hai-Ge Hou, Ting Gao, Jing-Lin Yang, Yu Yang

**Affiliations:** a School of Chemistry and Materials Science, Heilongjiang University, Harbin 150080, People’s Republic of China

## Abstract

In the title heterodinuclear complex, [CuNa(C_8_H_7_O_3_)_2_(NO_3_)]_*n*_, the Cu^II^ ion is five-coordinated in a square-pyramidal arrangement by four atoms of two different ligand molecules in equatorial positions and one remote nitrate O atom in the apical position. The Na^+^ ion is eight-coordinated by four ligand O atoms and four nitrate O atoms. The ligand links the Cu^II^ and Na ions, forming a layered arrangement extending parallel to (001).

## Related literature

For similar nickel–sodium complexes, see Costes *et al.* (1997*a* ▶,*b* ▶).
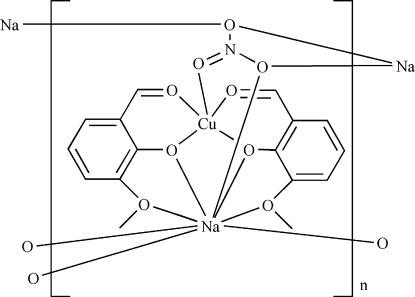


## Experimental

### 

#### Crystal data

[CuNa(C_8_H_7_O_3_)_2_(NO_3_)]
*M* *_r_* = 450.81
Orthorhombic, 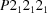

*a* = 7.737 (2) Å
*b* = 13.165 (4) Å
*c* = 16.889 (6) Å
*V* = 1720.2 (9) Å^3^
*Z* = 4
Mo *K*α radiation
μ = 1.35 mm^−1^
*T* = 293 K
0.35 × 0.33 × 0.30 mm

#### Data collection

Rigaku R-AXIS RAPID diffractometer
Absorption correction: multi-scan (*ABSCOR*; Higashi, 1995 ▶) *T*_min_ = 0.648, *T*_max_ = 0.691
16868 measured reflections
3926 independent reflections
3692 reflections with *I* > 2σ(*I*)
*R*_int_ = 0.025

#### Refinement

*R*[*F* ^2^ > 2σ(*F* ^2^)] = 0.021
*wR*(*F* ^2^) = 0.057
*S* = 1.03
3926 reflections
255 parameters
H-atom parameters constrained
Δρ_max_ = 0.22 e Å^−3^
Δρ_min_ = −0.21 e Å^−3^
Absolute structure: Flack (1983 ▶), 1671 Friedel pairs
Flack parameter: 0.007 (8)

### 

Data collection: *RAPID-AUTO* (Rigaku, 1998 ▶); cell refinement: *RAPID-AUTO*; data reduction: *CrystalClear* (Rigaku/MSC, 2002 ▶); program(s) used to solve structure: *SHELXS97* (Sheldrick, 2008 ▶); program(s) used to refine structure: *SHELXL97* (Sheldrick, 2008 ▶); molecular graphics: *SHELXTL* (Sheldrick, 2008 ▶); software used to prepare material for publication: *SHELXL97* .

## Supplementary Material

Crystal structure: contains datablock(s) global, I. DOI: 10.1107/S1600536811040025/ng5236sup1.cif

Structure factors: contains datablock(s) I. DOI: 10.1107/S1600536811040025/ng5236Isup2.hkl

Additional supplementary materials:  crystallographic information; 3D view; checkCIF report

## Figures and Tables

**Table 1:** Selected bond lengths (Å)

Cu1—O5	1.8989 (12)
Cu1—O2	1.9074 (12)
Cu1—O6	1.9487 (12)
Cu1—O3	1.9608 (14)
Cu1—O7	2.3560 (14)
Na1—O2	2.3667 (16)
Na1—O5	2.3900 (14)
Na1—O8^i^	2.4154 (17)
Na1—O1	2.5249 (15)
Na1—O4	2.6129 (16)
Na1—O9^i^	2.749 (2)
Na1—O9^ii^	2.755 (2)
Na1—O8	2.937 (2)

Symmetry codes: (i) 
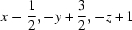
; (ii) 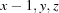.

**Fig. 1.**
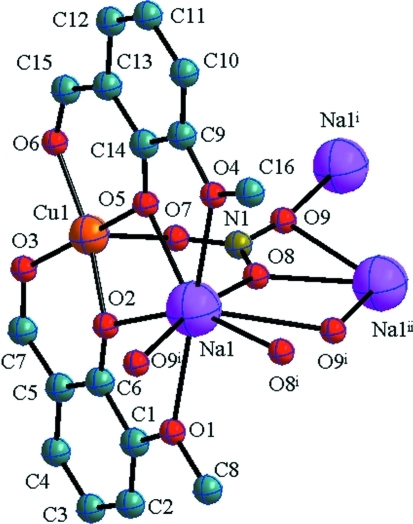
The molecular structure of the the title compound, showing 30% probability displacement ellipsoids.

## supplementary crystallographic information

### Comment

Orthovanillin is a commercial ligand that is able to chelate 3 d ions,mainly with copper ions. Furthermore, orthovanillin can also yield heterodinuclear 3 d-4f complexes and the first examples involve Cu—Ln complexes (Costes *et al.*, 1997*a*,*b*). We were interested in the nature of the products obtained by reacting a mononuclear 3 d complex with alkali metal ions. As shown in Fig. 1, The Cu^II^ is four-coordinated by two aldehyde oxygen atoms and two phenol oxygen atoms from the two orthovanillin ligands. The copper atom centre is inserted into the inner cavity. The Na ion is ligated by two hydroxyl oxygen atoms, two methoxyl oxygen atoms, two oxygen atoms of one bidentate nitrate counterion and two oxygen atoms of two different monodentate nitrate counterion. The Cu^II^ and Na are bridged by the phenolic oxygen atoms, layered arrangement extending parallel to (001).

### Experimental

To a solution of *o*-vanillin (0.046 g, 0.20 mmol) in dichloromethane (5 ml) was added to a solution of copper(II) acetate monohydrate (0.040 g, 0.20 mmol) and sodium nitrate (0.086 g, 0.20 mmol) in ethanol (5 ml). The mixture was stirred, heated under reflux (30 min) and then allowed to cool to room temperature. Yield: 70%. The crystals suitable for X-ray determination were obtained by slow diffusion of diethylether into the solution for one week. Analysis calculated for C_16_H_14_NCuNaO_9_: C 42.63, H 3.13, N 3.11%; Found: C 42.29, H 3.17, N, 3.22%.

### Refinement

H atoms bound to C atoms were placed in calculated positions and treated as riding on their parent atoms, with C—H = 0.93 Å (aromatic C), C—H = 0.97 Å (methylene C), and with *U*_iso_(H) = 1.2Ueq(C) or C—H = 0.96 Å (methly C) and with *U*_iso_(H) = 1.5Ueq(C).

### Crystal data

**Table:**

[CuNa(C_8_H_7_O_3_)_2_(NO_3_)]	*F*(000) = 916
*M**_r_* = 450.81	*D*_x_ = 1.741 Mg m^−^^3^
Orthorhombic, *P*2_1_2_1_2_1_	Mo *K*α radiation, λ = 0.71073 Å
Hall symbol: P 2ac 2ab	Cell parameters from 15663 reflections
*a* = 7.737 (2) Å	θ = 3.1–27.5°
*b* = 13.165 (4) Å	µ = 1.35 mm^−^^1^
*c* = 16.889 (6) Å	*T* = 293 K
*V* = 1720.2 (9) Å^3^	Block, brown
*Z* = 4	0.35 × 0.33 × 0.30 mm

### Data collection

**Table:**

Rigaku R-AXIS RAPID diffractometer	3926 independent reflections
Radiation source: fine-focus sealed tube	3692 reflections with *I* > 2σ(*I*)
graphite	*R*_int_ = 0.025
ω scans	θ_max_ = 27.5°, θ_min_ = 3.1°
Absorption correction: multi-scan (*ABSCOR*; Higashi, 1995)	*h* = −9→10
*T*_min_ = 0.648, *T*_max_ = 0.691	*k* = −17→16
16868 measured reflections	*l* = −21→21

### Refinement

**Table:**

Refinement on *F*^2^	Secondary atom site location: difference Fourier map
Least-squares matrix: full	Hydrogen site location: inferred from neighbouring sites
*R*[*F*^2^ > 2σ(*F*^2^)] = 0.021	H-atom parameters constrained
*wR*(*F*^2^) = 0.057	*w* = 1/[σ^2^(*F*_o_^2^) + (0.0375*P*)^2^] where *P* = (*F*_o_^2^ + 2*F*_c_^2^)/3
*S* = 1.03	(Δ/σ)_max_ = 0.001
3926 reflections	Δρ_max_ = 0.22 e Å^−^^3^
255 parameters	Δρ_min_ = −0.21 e Å^−^^3^
0 restraints	Absolute structure: Flack (1983), 1671 Friedel pairs
Primary atom site location: structure-invariant direct methods	Flack parameter: 0.007 (8)

### Special details

**Table:**

Geometry. All e.s.d.'s (except the e.s.d. in the dihedral angle between two l.s. planes) are estimated using the full covariance matrix. The cell e.s.d.'s are taken into account individually in the estimation of e.s.d.'s in distances, angles and torsion angles; correlations between e.s.d.'s in cell parameters are only used when they are defined by crystal symmetry. An approximate (isotropic) treatment of cell e.s.d.'s is used for estimating e.s.d.'s involving l.s. planes.
Refinement. Refinement of *F*^2^ against ALL reflections. The weighted *R*-factor *wR* and goodness of fit *S* are based on *F*^2^, conventional *R*-factors *R* are based on *F*, with *F* set to zero for negative *F*^2^. The threshold expression of *F*^2^ > σ(*F*^2^) is used only for calculating *R*-factors(gt) *etc*. and is not relevant to the choice of reflections for refinement. *R*-factors based on *F*^2^ are statistically about twice as large as those based on *F*, and *R*- factors based on ALL data will be even larger.

### Fractional atomic coordinates and isotropic or equivalent isotropic displacement parameters (Å^2^)

**Table:**

	*x*	*y*	*z*	*U*_iso_*/*U*_eq_	
C1	0.0679 (2)	0.92359 (12)	0.67688 (10)	0.0321 (3)	
C2	0.0859 (2)	1.02319 (13)	0.70061 (11)	0.0375 (4)	
H2	0.0378	1.0749	0.6703	0.045*	
C3	0.1765 (2)	1.04734 (13)	0.77039 (12)	0.0411 (4)	
H3	0.1895	1.1148	0.7856	0.049*	
C4	0.2450 (3)	0.97166 (13)	0.81555 (10)	0.0406 (4)	
H4	0.3025	0.9878	0.8622	0.049*	
C5	0.2298 (2)	0.86831 (12)	0.79230 (10)	0.0331 (3)	
C6	0.14000 (19)	0.84231 (11)	0.72185 (10)	0.0293 (3)	
C7	0.3021 (2)	0.79245 (15)	0.84132 (11)	0.0401 (4)	
H7	0.3452	0.8149	0.8897	0.048*	
C8	−0.0953 (3)	0.96653 (15)	0.56154 (13)	0.0484 (5)	
H8A	−0.1781	1.0034	0.5926	0.073*	
H8B	−0.1529	0.9341	0.5180	0.073*	
H8C	−0.0095	1.0126	0.5416	0.073*	
C9	0.0758 (2)	0.44760 (12)	0.54819 (9)	0.0309 (3)	
C10	0.0625 (3)	0.34687 (13)	0.52792 (12)	0.0421 (4)	
H10	0.0032	0.3281	0.4823	0.051*	
C11	0.1387 (3)	0.27166 (13)	0.57635 (13)	0.0495 (5)	
H11	0.1292	0.2036	0.5622	0.059*	
C12	0.2248 (3)	0.29714 (13)	0.64274 (12)	0.0446 (5)	
H12	0.2738	0.2466	0.6740	0.054*	
C13	0.2416 (3)	0.40139 (11)	0.66559 (10)	0.0337 (3)	
C14	0.1682 (2)	0.47851 (12)	0.61750 (9)	0.0287 (3)	
C15	0.3276 (2)	0.42379 (13)	0.73717 (11)	0.0379 (4)	
H15	0.3728	0.3688	0.7648	0.046*	
C16	−0.0964 (3)	0.50378 (17)	0.43943 (13)	0.0513 (5)	
H16A	−0.0241	0.4728	0.4000	0.077*	
H16B	−0.1458	0.5650	0.4185	0.077*	
H16C	−0.1872	0.4577	0.4538	0.077*	
Cu1	0.25742 (3)	0.636953 (13)	0.726538 (11)	0.03049 (6)	
N1	0.5362 (2)	0.72115 (11)	0.60308 (10)	0.0391 (3)	
Na1	0.03464 (14)	0.71081 (6)	0.56511 (5)	0.0556 (2)	
O1	−0.01427 (18)	0.89168 (9)	0.60950 (8)	0.0423 (3)	
O2	0.11760 (15)	0.74989 (9)	0.69651 (7)	0.0349 (3)	
O3	0.31612 (18)	0.69909 (10)	0.82857 (8)	0.0433 (3)	
O4	0.00468 (16)	0.52733 (9)	0.50775 (7)	0.0367 (3)	
O5	0.17851 (16)	0.57479 (8)	0.63174 (7)	0.0330 (2)	
O6	0.34982 (16)	0.50983 (9)	0.76779 (8)	0.0386 (3)	
O7	0.51745 (17)	0.70304 (11)	0.67537 (8)	0.0449 (3)	
O8	0.4100 (2)	0.74389 (12)	0.56117 (8)	0.0547 (4)	
O9	0.6790 (2)	0.7162 (2)	0.57232 (13)	0.0937 (7)	

### Atomic displacement parameters (Å^2^)

**Table:**

	*U*^11^	*U*^22^	*U*^33^	*U*^12^	*U*^13^	*U*^23^
C1	0.0297 (8)	0.0350 (8)	0.0315 (8)	0.0009 (7)	0.0035 (7)	−0.0002 (7)
C2	0.0381 (9)	0.0324 (8)	0.0421 (10)	0.0028 (7)	0.0080 (7)	0.0033 (7)
C3	0.0414 (9)	0.0336 (8)	0.0482 (10)	−0.0039 (7)	0.0074 (9)	−0.0076 (8)
C4	0.0388 (9)	0.0458 (9)	0.0373 (8)	−0.0037 (10)	0.0026 (9)	−0.0099 (7)
C5	0.0318 (8)	0.0379 (8)	0.0296 (7)	0.0008 (8)	0.0032 (6)	−0.0020 (6)
C6	0.0266 (7)	0.0320 (7)	0.0294 (7)	0.0012 (6)	0.0037 (6)	−0.0007 (6)
C7	0.0437 (10)	0.0507 (9)	0.0258 (8)	0.0024 (8)	−0.0044 (7)	−0.0043 (7)
C8	0.0524 (12)	0.0468 (10)	0.0461 (11)	0.0120 (10)	−0.0117 (9)	0.0078 (9)
C9	0.0315 (8)	0.0313 (7)	0.0299 (8)	0.0006 (7)	0.0048 (7)	0.0017 (6)
C10	0.0514 (10)	0.0351 (8)	0.0399 (9)	−0.0018 (8)	0.0041 (8)	−0.0086 (8)
C11	0.0703 (14)	0.0269 (7)	0.0513 (12)	0.0020 (9)	0.0068 (11)	−0.0041 (8)
C12	0.0541 (12)	0.0288 (7)	0.0508 (10)	0.0105 (9)	0.0092 (10)	0.0069 (7)
C13	0.0352 (8)	0.0291 (6)	0.0367 (8)	0.0024 (8)	0.0045 (8)	0.0056 (6)
C14	0.0289 (7)	0.0278 (7)	0.0293 (8)	0.0006 (6)	0.0053 (6)	0.0029 (6)
C15	0.0399 (9)	0.0345 (8)	0.0394 (10)	0.0085 (8)	0.0024 (8)	0.0116 (7)
C16	0.0540 (12)	0.0548 (11)	0.0453 (11)	−0.0040 (10)	−0.0180 (10)	−0.0021 (9)
Cu1	0.03520 (10)	0.02981 (9)	0.02645 (10)	0.00292 (9)	−0.00451 (9)	0.00259 (7)
N1	0.0461 (9)	0.0354 (7)	0.0357 (8)	−0.0090 (7)	0.0044 (7)	0.0047 (6)
Na1	0.0954 (7)	0.0374 (4)	0.0340 (4)	0.0132 (4)	−0.0188 (4)	0.0012 (3)
O1	0.0518 (7)	0.0344 (6)	0.0407 (7)	0.0082 (6)	−0.0130 (6)	0.0016 (5)
O2	0.0404 (6)	0.0302 (5)	0.0340 (6)	0.0051 (5)	−0.0081 (5)	−0.0019 (5)
O3	0.0581 (8)	0.0437 (6)	0.0282 (6)	0.0092 (6)	−0.0082 (6)	0.0006 (5)
O4	0.0423 (7)	0.0344 (6)	0.0334 (6)	0.0006 (6)	−0.0082 (5)	0.0009 (5)
O5	0.0434 (6)	0.0244 (5)	0.0312 (6)	−0.0007 (5)	−0.0066 (5)	0.0042 (4)
O6	0.0415 (6)	0.0402 (6)	0.0343 (6)	0.0054 (5)	−0.0075 (6)	0.0078 (5)
O7	0.0448 (7)	0.0593 (8)	0.0305 (6)	−0.0087 (6)	−0.0033 (6)	0.0084 (6)
O8	0.0627 (9)	0.0642 (9)	0.0373 (8)	−0.0019 (8)	−0.0063 (7)	0.0162 (7)
O9	0.0520 (10)	0.158 (2)	0.0716 (13)	−0.0040 (12)	0.0224 (9)	0.0250 (14)

### Geometric parameters (Å, °)

**Table:**

C1—O1	1.370 (2)	C14—O5	1.2925 (19)
C1—C2	1.378 (2)	C15—O6	1.257 (2)
C1—C6	1.426 (2)	C15—H15	0.9300
C2—C3	1.407 (3)	C16—O4	1.428 (2)
C2—H2	0.9300	C16—H16A	0.9600
C3—C4	1.362 (3)	C16—H16B	0.9600
C3—H3	0.9300	C16—H16C	0.9600
C4—C5	1.421 (2)	Cu1—O5	1.8989 (12)
C4—H4	0.9300	Cu1—O2	1.9074 (12)
C5—C7	1.413 (2)	Cu1—O6	1.9487 (12)
C5—C6	1.420 (2)	Cu1—O3	1.9608 (14)
C6—O2	1.3013 (19)	Cu1—O7	2.3560 (14)
C7—O3	1.252 (2)	Cu1—Na1	3.3688 (11)
C7—H7	0.9300	N1—O9	1.223 (2)
C8—O1	1.421 (2)	N1—O8	1.243 (2)
C8—H8A	0.9600	N1—O7	1.252 (2)
C8—H8B	0.9600	N1—Na1^i^	2.978 (2)
C8—H8C	0.9600	Na1—O2	2.3667 (16)
C9—O4	1.368 (2)	Na1—O5	2.3900 (14)
C9—C10	1.373 (2)	Na1—O8^ii^	2.4154 (17)
C9—C14	1.431 (2)	Na1—O1	2.5249 (15)
C10—C11	1.413 (3)	Na1—O4	2.6129 (16)
C10—H10	0.9300	Na1—O9^ii^	2.749 (2)
C11—C12	1.347 (3)	Na1—O9^iii^	2.755 (2)
C11—H11	0.9300	Na1—O8	2.937 (2)
C12—C13	1.431 (2)	Na1—N1^ii^	2.978 (2)
C12—H12	0.9300	O8—Na1^i^	2.4154 (17)
C13—C15	1.411 (3)	O9—Na1^i^	2.749 (2)
C13—C14	1.419 (2)	O9—Na1^iv^	2.755 (2)
			
O1—C1—C2	125.48 (15)	O8—N1—O7	120.68 (16)
O1—C1—C6	113.20 (14)	O9—N1—Na1^i^	67.31 (13)
C2—C1—C6	121.31 (16)	O8—N1—Na1^i^	51.77 (9)
C1—C2—C3	120.59 (16)	O7—N1—Na1^i^	170.58 (13)
C1—C2—H2	119.7	O2—Na1—O5	66.12 (4)
C3—C2—H2	119.7	O2—Na1—O8^ii^	151.95 (6)
C4—C3—C2	119.82 (16)	O5—Na1—O8^ii^	141.87 (6)
C4—C3—H3	120.1	O2—Na1—O1	63.72 (4)
C2—C3—H3	120.1	O5—Na1—O1	129.54 (5)
C3—C4—C5	120.89 (17)	O8^ii^—Na1—O1	88.25 (6)
C3—C4—H4	119.6	O2—Na1—O4	124.94 (5)
C5—C4—H4	119.6	O5—Na1—O4	61.52 (4)
C7—C5—C6	120.96 (15)	O8^ii^—Na1—O4	82.27 (5)
C7—C5—C4	118.82 (16)	O1—Na1—O4	165.61 (6)
C6—C5—C4	120.21 (15)	O2—Na1—O9^ii^	127.27 (7)
O2—C6—C5	124.49 (14)	O5—Na1—O9^ii^	118.04 (7)
O2—C6—C1	118.32 (15)	O8^ii^—Na1—O9^ii^	47.97 (5)
C5—C6—C1	117.18 (14)	O1—Na1—O9^ii^	88.98 (7)
O3—C7—C5	128.84 (17)	O4—Na1—O9^ii^	92.63 (7)
O3—C7—H7	115.6	O2—Na1—O9^iii^	102.93 (7)
C5—C7—H7	115.6	O5—Na1—O9^iii^	117.63 (7)
O1—C8—H8A	109.5	O8^ii^—Na1—O9^iii^	68.52 (6)
O1—C8—H8B	109.5	O1—Na1—O9^iii^	79.21 (7)
H8A—C8—H8B	109.5	O4—Na1—O9^iii^	87.24 (7)
O1—C8—H8C	109.5	O9^ii^—Na1—O9^iii^	115.72 (6)
H8A—C8—H8C	109.5	O2—Na1—O8	73.79 (5)
H8B—C8—H8C	109.5	O5—Na1—O8	70.20 (5)
O4—C9—C10	125.88 (16)	O8^ii^—Na1—O8	109.77 (5)
O4—C9—C14	113.03 (14)	O1—Na1—O8	90.86 (5)
C10—C9—C14	121.08 (16)	O4—Na1—O8	102.50 (6)
C9—C10—C11	120.06 (18)	O9^ii^—Na1—O8	61.80 (5)
C9—C10—H10	120.0	O9^iii^—Na1—O8	169.91 (7)
C11—C10—H10	120.0	O2—Na1—N1^ii^	145.87 (6)
C12—C11—C10	120.92 (16)	O5—Na1—N1^ii^	132.26 (6)
C12—C11—H11	119.5	O8^ii^—Na1—N1^ii^	23.83 (5)
C10—C11—H11	119.5	O1—Na1—N1^ii^	90.01 (5)
C11—C12—C13	120.54 (17)	O4—Na1—N1^ii^	85.69 (5)
C11—C12—H12	119.7	O9^ii^—Na1—N1^ii^	24.23 (5)
C13—C12—H12	119.7	O9^iii^—Na1—N1^ii^	92.20 (6)
C15—C13—C14	121.97 (15)	O8—Na1—N1^ii^	85.98 (5)
C15—C13—C12	118.31 (16)	O2—Na1—Cu1	33.40 (3)
C14—C13—C12	119.70 (16)	O5—Na1—Cu1	33.32 (3)
O5—C14—C13	124.79 (15)	O8^ii^—Na1—Cu1	171.94 (5)
O5—C14—C9	117.52 (14)	O1—Na1—Cu1	96.23 (4)
C13—C14—C9	117.69 (15)	O4—Na1—Cu1	94.51 (4)
O6—C15—C13	127.26 (15)	O9^ii^—Na1—Cu1	125.19 (5)
O6—C15—H15	116.4	O9^iii^—Na1—Cu1	118.86 (6)
C13—C15—H15	116.4	O8—Na1—Cu1	63.60 (3)
O4—C16—H16A	109.5	N1^ii^—Na1—Cu1	148.94 (5)
O4—C16—H16B	109.5	C1—O1—C8	117.75 (14)
H16A—C16—H16B	109.5	C1—O1—Na1	117.80 (10)
O4—C16—H16C	109.5	C8—O1—Na1	123.41 (12)
H16A—C16—H16C	109.5	C6—O2—Cu1	124.46 (10)
H16B—C16—H16C	109.5	C6—O2—Na1	123.21 (11)
O5—Cu1—O2	85.95 (5)	Cu1—O2—Na1	103.51 (5)
O5—Cu1—O6	92.82 (5)	C7—O3—Cu1	122.72 (12)
O2—Cu1—O6	166.56 (5)	C9—O4—C16	117.21 (14)
O5—Cu1—O3	174.40 (6)	C9—O4—Na1	119.25 (10)
O2—Cu1—O3	92.28 (5)	C16—O4—Na1	123.29 (11)
O6—Cu1—O3	87.66 (6)	C14—O5—Cu1	126.82 (10)
O5—Cu1—O7	97.15 (5)	C14—O5—Na1	128.20 (11)
O2—Cu1—O7	95.67 (5)	Cu1—O5—Na1	102.93 (5)
O6—Cu1—O7	97.76 (5)	C15—O6—Cu1	125.21 (11)
O3—Cu1—O7	88.31 (6)	N1—O7—Cu1	121.78 (11)
O5—Cu1—Na1	43.75 (4)	N1—O8—Na1^i^	104.40 (12)
O2—Cu1—Na1	43.08 (4)	N1—O8—Na1	136.74 (11)
O6—Cu1—Na1	136.46 (4)	Na1^i^—O8—Na1	116.83 (6)
O3—Cu1—Na1	135.18 (4)	N1—O9—Na1^i^	88.46 (14)
O7—Cu1—Na1	91.92 (4)	N1—O9—Na1^iv^	157.32 (17)
O9—N1—O8	118.74 (18)	Na1^i^—O9—Na1^iv^	112.18 (7)
O9—N1—O7	120.58 (18)		

Symmetry codes: (i) *x*+1/2, −*y*+3/2, −*z*+1; (ii) *x*−1/2, −*y*+3/2, −*z*+1; (iii) *x*−1, *y*, *z*; (iv) *x*+1, *y*, *z*.
